# Decoupling Internalized Dysfunctional Attachments: A Combined ACT and Schema Therapy Approach

**DOI:** 10.3389/fpsyg.2018.02332

**Published:** 2018-11-23

**Authors:** Alessandro Grecucci, Irene Messina, Harold Dadomo

**Affiliations:** ^1^Department of Psychology and Cognitive Sciences, University of Trento, Trento, Italy; ^2^Department of Psychiatry and Psychotherapy III, University of Ulm, Ulm, Germany; ^3^Department of Psychology, Universitas Mercatorum, Rome, Italy; ^4^Unity of Neuroscience, Department of Medicine and Surgery, University of Parma, Parma, Italy

**Keywords:** Schema therapy, mindfulness, emotion regulation, attachment, parenting

In the field of attachment research, parental behavior has been described in terms of caregiving behavioral systems, expressed in specific relational patterns. These patterns are meant to provide assurance and comfort for the infants to promote exploration and autonomy by ensuring a “secure base” to return to Bowlby (1988). In case of stressful situations for the child, the prototypical behavior of caregivers is to ensure protection and responding with emphatic emotions. However, not every parent is equally skilled and motivated to be an effective caregiver and individuals may differ in their caregiving-related behaviors. In some cases, caregivers are not able to provide protection for their child and/or they may respond with dysregulated emotions.

According to attachment theory, such individual differences can be attributed to *mental representation* of the self and the others (or *Internal Working Models* as Bowlby defined them). Caregivers' mental representations of their child and of the self as caregiver influence their behavior, leading to different relational patters with their children (Solomon and George, 2008). For instance, George and Solomon (1989, 1996), showed that mothers of secure children have positive representations of the self as caregiver as they perceive themselves as effective and caring, together with a positive and realistic perception of their child. In contrast, mothers of avoidant children have more negative representations of the self as caregiver, and they tend to devalue their child's attachment needs. Mothers of ambivalent children are characterized by uncertainty and confusion, and they tend to promote the dependency of their children. Last, but not least, mothers of disorganized children abdicate caregiving considering themselves as helpless and unable to protect their child (George and Solomon, 1996). Caregivers' mental representations is a very critical aspect in parent-infant relationship, because internalized dysfunctional attachments in parents (in the form of representations) may generate negative expectations and emotions about self and others, and drive maladaptive coping responses (McKay et al., 2012). Unfortunately, these mental representations are transmitted from one generation to the other (Bretherton, 1990). In this paper, we suggest a methodology to stop the repetition of past dysfunctional relational patterns, and assist the parent in developing new caregiving abilities.

Third wave psychotherapy approaches offer interesting tools for the intervention on internal representations. For instance, these approaches can limit the influence of negative past representations toward significant others in current relationships (Simeone-DiFrancesco et al., 2015). We make the point that this consideration applies to parenting style as well. We propose a two-steps procedure based on the integration of Schema Therapy (ST; Loose et al., 2013; Young et al., 2013; Simeone-DiFrancesco et al., 2015), and techniques derived from Mindfulness (Van Vreeswijk et al., 2016), and Acceptance and Commitment Therapy (ACT; Hayes et al., 1999). This integration stems from the fact that these approaches have, in our opinion, complementary strengths. On one side, ST provides the conceptual background for an understanding of what is enacted in the current relationship, say a Dysfunctional Parent Mode (e.g., Punitive, Demanding, Critic parent…), coming from past internalized relationships. Therapists may refer to the concept of *enactment of specific Modes* to help caregivers become aware of roles and relational themes displayed during the interactions with their children. ACT and mindfulness, on the other hand, have outlined a series of strategies by which clinicians can help the client to attend, observe and stop automatic reactions to dysfunctional mental representations. A combined ST and ACT approach aims to relieve parenting-related difficulties, through the understanding and the limitation of enactments in the parent-child relationship. Previous contributions have considered the integration between ST and ACT or mindfulness approaches, without a specific focus on parental skills (see for example the integration of Schema therapy with mindfulness by Van Vreeswijk et al. (2016); or the incorporation of acceptance-based concepts into Schema therapy by Farrell and Shaw (2017)). These approaches outline effective methods to manage emotional experience in the present moment and to reduce reactivity to dysfunctional emotional schemas (Van Vreeswijk et al., 2016). Moreover, Loose and colleagues (Loose et al., 2013) have proposed a modified version of Schema Therapy (ST), specific for children and adolescents. Although parents are sometimes involved during the therapy, this model focus on children, whereas the issue of helping parenting is only briefly discussed. In contrast to such mentioned models, our discussion is specifically focused on parents, and integrate ST and other third wave approaches.

The first step of the methodology we propose is pathological modes identification. In Schema Therapy terminology, internalized dysfunctional attachments can be described as coupled *Dysfunctional-child-modes* (DCM, for example, Angry Child, Impulsive Child), with *Dysfunctional-parent-modes* (DPM, for example, Punitive Parent, Demanding Parent, Critic Parent), eventually managed with *Dysfunctional-coping-strategies* (DCS, for example, detached protector, overcompensating modes, or compliant surrender modes). In daily life, the activation of dysfunctional modes negatively influences parents' attitudes and behaviors toward their child. This is because DCM and DPM are associated with either (1) dysregulated emotions, or with (2) dysregulatory mechanisms (Dadomo et al., 2016, 2018). DPM are the primary source of dysregulated emotions and reflect pathological aspects of the parent that are enacted inside the relationship. In terms of emotion regulation, these DPM are dysregulatory mechanisms that generate the most severe dysregulated emotions in the parent (for example, a Punitive Parent Mode that induces in the child self-hate and contempt toward the self). DCM, as a consequence, are characterized by specific dysregulated emotions (Angry Child = anger, Lonely Child = sadness, Anxious Child = fear). DCM develop when certain basic emotional needs are not adequately met in childhood. To complete the picture, DCS are pathological *regulatory mechanisms* that paradoxically increase dysregulation in the long run.

Based on concepts derived from Schema Therapy (Loose et al., 2013; Young et al., 2013; Simeone-DiFrancesco et al., 2015; Van Vreeswijk et al., 2016), step 1 helps parents become aware of their internal representations (DPM-DCM), and the pathological strategies they use (DCS) when they activate. This can be achieved as follows:

- Detection of pathological modes by interviewing parents, or by using self-administered questionnaires (Young Parenting Inventory, Schema Mode Inventory, etc). This is the first step to make explicit relational patterns that are driving dysfunctional parent modes and causing distress to their children.
- Psychoeducation on specific DPM-DCM, providing information concerning how individuals enact past patterns into their parental relationships. Examination of specific daily life examples may be useful at this step. This may help clients develop awareness on how they enact problematic ways of relating to their children.
- Examination of the negative consequences of enacting these modes (DCS) into the parent-child relationship. With this aim, chair work with an empty chair representing the child may promote parents' awareness of DCS and their consequences for the child.
- Identification of values. This aspect regards the clarification of the kind of parent our patient wants to become. Since values drive patients' behaviors, such clarification may provide a guide and motivation to try new responses. The work on values can also help modifying internal mental representations of the self as parent.

At the end of this phase, we expect the client to be much more aware of her/his DPM. However, an additional phase to reduce DCS and to develop new strategies is necessary. With this aim, we now turn to step 2, taking into consideration third wave cognitive therapies. In step 2, the clinician helps the parent to reduce DCS by using mindfulness and ACT to reduce over-reactivity. We suggest the following steps, as a methodology to promote a change regarding how parents relate with their modes:

- Mindful exposition and observation (without acting) of DPM and the emotions and action tendencies associated. This encourages parents to attend and reflect on pathological experience. Simulations and imagery techniques can greatly help the parent to activate DCS.
- Once activated, exposure plus a non-judgmental stance as prescribed by mindfulness and ACT theorists, may help containing the emotional experience. Acceptance and non-reactivity attitude rather than enacting the DCS in response to the child's needs is fundamental to break the cycle. This aspect helps parents in creating a place to reflect on their automatic reactions without enacting them. Research suggests that acceptance decreases avoidance and increases valued actions (Twohig, 2007).
- Take the distance from modes. Techniques developed by ACT are useful to take distance from DPM. An example is defusion, a technique based on “looking at thoughts rather than from thoughts.” This may greatly help parents to disengage from automatic responses (DCS).
- Valued and committed actions application (according to values as proposed by ACT). Committed actions are values-based actions that may replace old DCS. This final part is the heart of step 2 and it help parents in developing new relational patterns. Chair work and simulation can be very helpful for the development of new relational patterns and for the exploration of positive effects of new behaviors (such as, more caring and protective behaviors in parent-infant relationship).

In sum, in the present opinion paper, we provided insights and technical advice on how to help parents to become aware of and to detach from dysfunctional ways of relating with their children. Although we do not have yet empirical data to support our model, we believe it is a promising approach to help parenting, as it is strongly grounded in therapy models whose efficacy has been demonstrated (see for example, Hacker et al., 2016; Taylor et al., 2017). Moreover, some preliminary studies on the integration between ST and ACT or mindfulness-based approaches (Amaro et al., 2010; Gojani et al., 2017), suggesting that the approach described here are probably efficacious.

Specifically, Amaro et al. (2010), designed a Spiritual Self-Schema as an 8-session mindfulness-based intervention to target addiction problems and human immunodeficiency virus (HIV) risk. Preliminary results showed high rates of Spiritual Self-Schema acceptability and positive changes in a number of outcomes relevant to recovery from addiction and to HIV prevention. More recently, Gojani et al. (2017) demonstrated similar and synergic effects of both schema- and mindfulness-based therapies on maladaptive schemas in improving the psoriasis patients with the psychopathologic symptoms. Future studies are needed to provide efficacy data on this combined ST and ACT approach for improving parenting.

## Author contributions

All authors listed have made a substantial, direct and intellectual contribution to the work, and approved it for publication.

### Conflict of interest statement

The authors declare that the research was conducted in the absence of any commercial or financial relationships that could be construed as a potential conflict of interest.

## References

[B1] Amaro H. Magno-Gatmaytan C. Meléndez M. Cortés D. E. Arevalo S. Margolin A. (2010). Addiction treatment intervention: an uncontrolled prospective pilot study of Spiritual Self-Schema therapy with Latina women. Subst. Abus. 31, 117–125. 10.1080/08897071003641602 20408063

[B2] Bowlby J. (1988). A Secure Base: Parent-Child Attachment and Healthy Human Development. New York, NY: Basic Books.

[B3] Bretherton I. (1990). Communication patterns, internal working models, and the intergenerational transmission of attachment relationships. Infant Ment. Health J. 11, 237–252.

[B4] Dadomo H. Grecucci A. Giardini I. Ugolini E. Carmelita A. Panzeri M. (2016). Schema therapy for emotional dysregulation: theoretical implication and clinical applications. Front. Psychol. 7:1987 10.3389/fpsyg.2016.01987 28066304 PMC5177643

[B5] Dadomo H. Panzeri M. Caponcello D. Carmelita A. Grecucci A. (2018). Schema therapy for emotional dysregulation in personality disorders: a review. Curr. Opin. Psychiatry 31, 43–49. 10.1097/YCO.0000000000000380 29120915

[B6] Farrell J. M. Shaw I. A. (2017). Experiencing Schema Therapy from the Inside Out a Self-Practice/Self-Reflection Workbook for Therapists. New York, NY: The Guilford Press.

[B7] George C. Solomon J. (1989). Internal working models of caregiving and security of attachment at age six. Infant Ment. Health J. 10, 222–237.

[B8] George C. Solomon J. (1996). Representational models of relationships: links between caregiving and attachment. Infant Ment. Health J. 17, 18–36.

[B9] Gojani P. J. Masjedi M. Khaleghipour S. Behzadi E. (2017). Effects of the schema therapy and mindfulness on the maladaptive schemas hold by the psoriasis patients with the psychopathology symptoms. Adv. Biomed. Res. 6:4. 10.4103/2277-9175.190988 28217649 PMC5309440

[B10] Hacker T. Stone P. MacBeth A. (2016). Acceptance and commitment therapy - Do we know enough? Cumulative and sequential meta-analyses of randomized controlled trials. J. Affect. Disord. 190, 551–565. 10.1016/j.jad.2015.10.053 26571105

[B11] Hayes S. C. Strosahl K. D. Wilson K. G. (1999). Acceptance and Commitment Therapy: An Experiential Approach to Behavior Change. New York, NY: The Guilford Press.

[B12] Loose C. Graaf P. Zarbock G. (2013). Schematherapie mit Kindern und Jugendlichen: Mit Online-Materialien. Beltz; Auflage: Originalausgabe.

[B13] McKay M. Lev A. Skeen M. (2012). Acceptance and Commitment Therapy for Inter- Personal Problems: Using Mindfulness, Acceptance, and Schema Awareness to Change Interpersonal Behaviors. Oakland, CA: New Harbinger Publications.

[B14] Simeone-DiFrancesco C. Roediger E. Stevens B. A. (2015). Schema Therapy With Couples: A Practitioner's Guide to Healing Relationships. New York, NY: Wiley-Blackwell.

[B15] Solomon J. George C. (2008). “The measurement of attachment security and related constructs in infancy and early childhood,” in Handbook of Attachment: Theory, Research, and Clinical Applications, eds Cassidy J. Shaver P. R (New York, NY: Guilford Press), 383–416.

[B16] Taylor C. D. J. Bee P. Haddock G. (2017). Does schema therapy change schemas and symptoms? A systematic review across mental health disorders. Psychol. Psychother. 90, 456–479. 10.1111/papt.12112 28035734 PMC5573974

[B17] Twohig M. (2007). A Randomized Clinical Trial of Acceptance and Commitment Therapy Versus Progressive Relaxation Training in the Treatment of Obsessive Compulsive Disorder. Ph.D. dissertation. University of Nevada, Reno. 10.1037/a0020508 PMC2948415 20873905

[B18] Van Vreeswijk M. Broersen J. Schurink G. (2016). Mindfulness and Schema Therapy: A Practical Guide. New York, NY: Wiley-Blackwell.

[B19] Young J. Klosko J. S. Weishaar M. E. (2013). Schema Therapy: A Practitioner's Guide. New York, NY: The Guilford Press.

